# Hard times in the city – attractive nest sites but insufficient food supply lead to low reproduction rates in a bird of prey

**DOI:** 10.1186/1742-9994-11-48

**Published:** 2014-05-27

**Authors:** Petra Sumasgutner, Erwin Nemeth, Graham Tebb, Harald W Krenn, Anita Gamauf

**Affiliations:** 1 Department of Integrative Zoology, University of Vienna, Althanstraße 14, Vienna A-1090, Austria; 2 1st Zoological Department, Museum of Natural History Vienna, Burgring 7, Vienna A-1010, Austria; 3 Max Planck Institute for Ornithology, Communication and Social Behaviour Group, Eberhard Gwinner-Straße, Seewiesen D-82319, Germany; 4 BirdLife Austria, Museumsplatz 1/10/8, Vienna A-1070, Austria; 5 University of Veterinary Medicine, Veterinärplatz 1, Vienna A-1210, Austria

**Keywords:** Diet choice, Ecological trap, *Falco tinnunculus*, Historical building structure, Nest site choice, Nest survival, Prey availability, Urban exploiter, Urban gradient

## Abstract

**Introduction:**

Urbanization is a global phenomenon that is encroaching on natural habitats and decreasing biodiversity, although it is creating new habitats for some species. The Eurasian kestrel (*Falco tinnunculus*) is frequently associated with urbanized landscapes but it is unclear what lies behind the high densities of kestrels in the urban environment.

**Results:**

Occupied nest sites in the city of Vienna, Austria were investigated along a gradient of urbanization (percentage of land covered by buildings or used by traffic). Field surveys determined the abundance of potential prey (birds and rodents) and the results were compared to the birds’ diets. A number of breeding parameters were recorded over the course of three years. The majority of kestrels breed in semi-natural cavities in historic buildings. Nearest neighbour distances (NND) were smallest and reproductive success lowest in the city centre. Abundance of potential prey was not found to relate to the degree of urbanization but there was a significant shift in the birds’ diets from a heavy reliance on rodents in the outskirts of the city to feeding more on small birds in the centre. The use of urban habitats was associated with higher nest failure, partly associated with predation and nest desertion, and with significantly lower hatching rates and smaller fledged broods.

**Conclusions:**

High breeding densities in urban habitats do not necessarily correlate with high habitat quality. The high density of kestrel nests in the city centre is probably due to the ready availability of breeding cavities. Highly urbanized areas in Vienna are associated with unexpected costs for the city dwelling-raptor, in terms both of prey availability and of reproductive success. The kestrel appears to be exploiting the urban environment but given the poor reproductive performance of urban kestrels it is likely that the species is falling into an ecological trap.

## Introduction

Rapidly increasing urbanization is a global phenomenon that affects not only humans but also animals and plants [1]. While native biodiversity often declines [2], urbanization promotes the biotic homogenization of species assemblages [3-5]. Because of the loss of natural habitat, urbanization generally leads to a complete restructuring of vegetation and species composition and has thus become a major concern in conservation biology [6,7].

The urban environment can induce dramatic changes in animal behaviour, physiology and life-history [8-11]. Within species, studies on passerines have shown that urban individuals have smaller clutches that are generally laid earlier and that their nestlings are lighter than those of their rural conspecifics [12]. Ultimately, species able to adapt to the challenges posed by increasing urbanization will persist and may even increase, while those that cannot will decline or disappear. Urbanization thus filters bird communities (review in [13]).

The success of urban species appears to be a function of the time since they initially colonized urban areas [14]. The most highly urbanized areas are dominated by ‘urban exploiters’ ([15,16]), a small number of mainly non-native species, especially nearctic passerines [17], whose success in urban areas is largely related to their ability to exploit human resources such as garbage dumps, feeders and nest boxes [18]. Many other species are also found in the centres of large cities, although it is often hard to determine whether they are benefitting or suffering from the urban environment. It is conceivable that the decision to breed in highly urbanized areas might be based on a mistaken assessment of the quality of the environment, with individuals in urban centres suffering from a lower availability of food and lower breeding success. In such cases, the species is said to have fallen into an ‘ecological trap’ [19].

The Eurasian kestrel (*Falco tinnunculus* Linnaeus, 1758) is clearly affected by urbanization. It was first recorded breeding in urban environments in the latter half of the 19th century [20] and is now commonly associated with urbanized landscapes [21]. A number of studies have been performed on the diet and breeding success of urban kestrels [22-27] but it is difficult to draw general conclusions from them, as each metropolis provides a unique habitat, differing from others in terms of size [28], building structure [29] and composition of vegetation [30,31]. Despite the previous work, it is still unclear whether the kestrel is a true urban exploiter or whether instead the urban environment represents an ecological trap for the species. The issue can best be addressed by analysing the breeding success of members of an urban population that is sufficiently large to permit the comparison between ‘city-dwellers’ and birds living in the suburbs.

The urban study area in Vienna (243 km^2^), Austria has the highest documented density of Eurasian kestrels in a non-colonial urban breeding population ([32,33], c.f. [22-27]) and is ideally suited to a study of this kind. We compared the species’ biology along an urban gradient, defined by the density of buildings and areas used by traffic [34]. We considered (1) whether the breeding density of kestrels in urbanized landscapes results mainly from the availability of nest sites, based on the historical building structure and asked (2) whether the use of the urban habitat is associated with differences in annual reproductive rates or (3) a sex bias in nestling survival. We also (4) analysed causes of nest failure and tested whether (5) there is a link between breeding density, reproductive success and availability of prey. Because of the data structure and the relatively small sample size, we pooled the nests investigated more closely into three defined urban zones, using the different zones as discrete explanatory variables (6) to examine the main categories of prey in the kestrels’ diet and (7) to relate the diet to the availability of prey.

## Results

### Nest site choice and nest site availability

**Figure 1:**
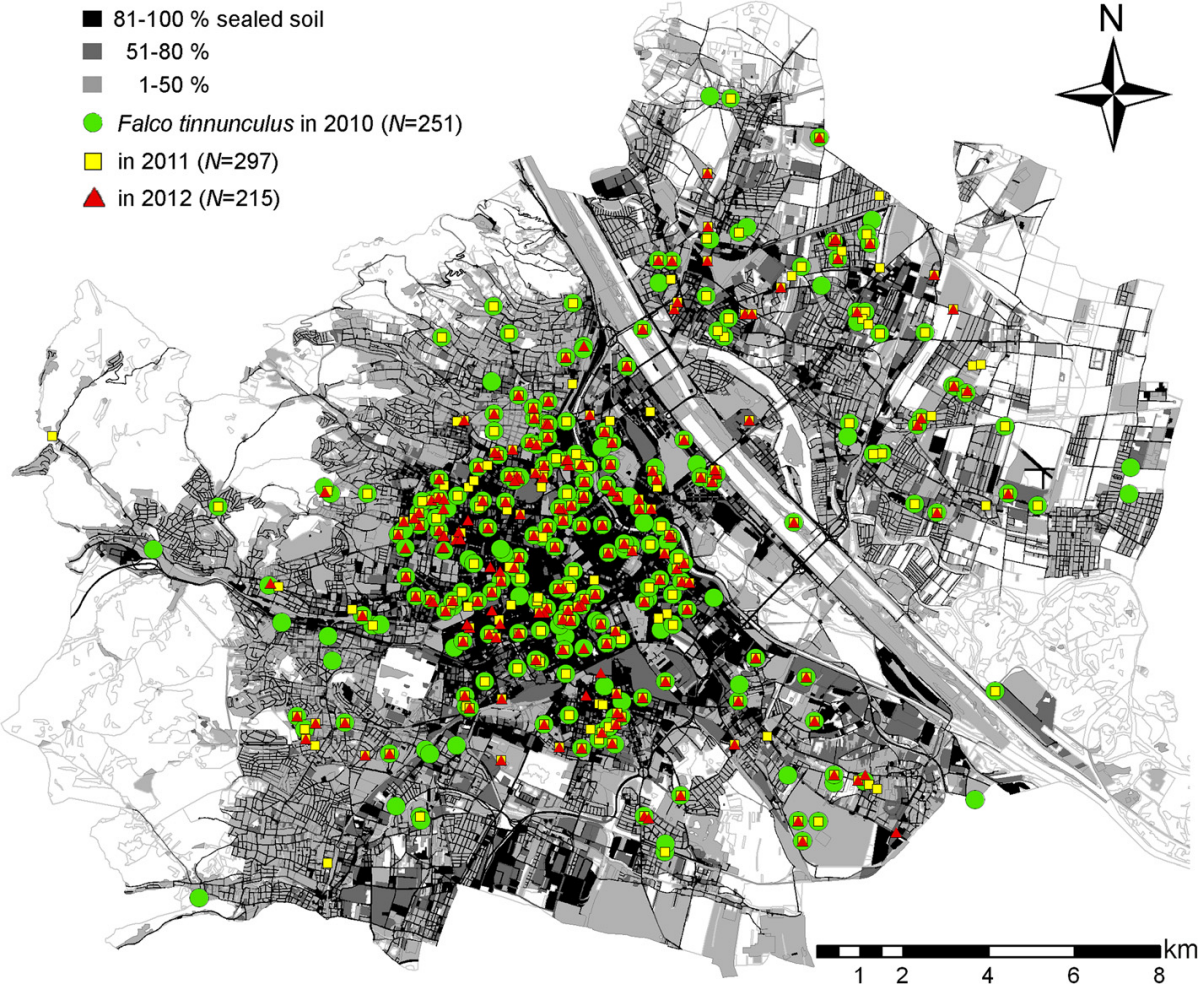
**Urban study area (243 km**^**2**^**) in Vienna, Austria.** The urban gradient, displayed from black to grey (white - unsealed soil outside the study area), and occupied nest sites of *Falco tinnunculus* during the study period (2010 – 2012).

The kestrel monitoring in 2010 found a total of 251 occupied nests, while in 2011 297 nests and in 2012 215 nests were found (Figure 1). The figures translate to a breeding density of 89–122 breeding pairs per 100 km^2^ in urbanized areas of Vienna. Kestrels predominantly breed in building cavities (69%, based on nests occupied in 2010), where they largely use roof openings (41%). Abandoned crow nests in trees are less frequently used (18% of broods). In rare cases, nest boxes (6%; 33 nest boxes were offered in the city) or window boxes (4%) are used.

**Figure 2:**
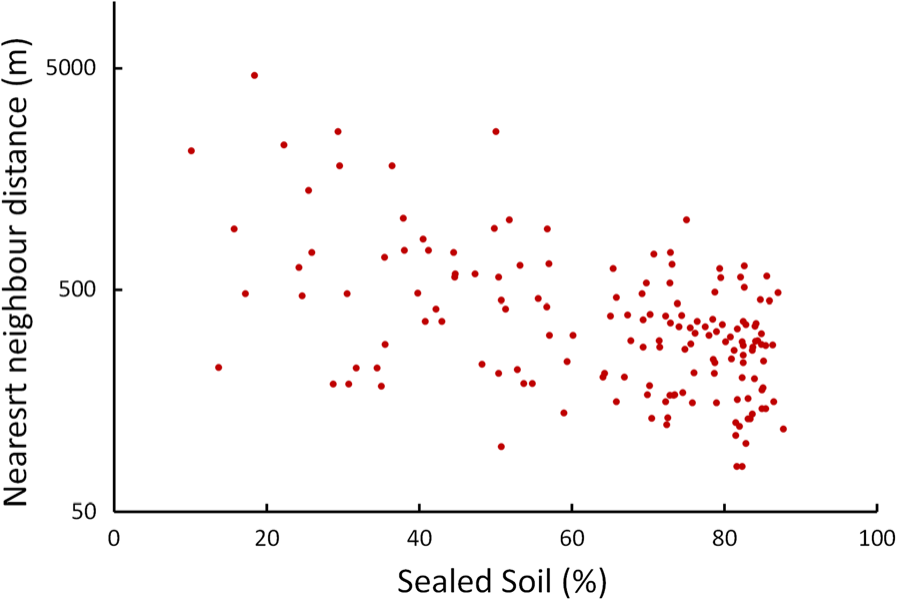
**Sealed soil (%) and nearest neighbour distance (NND) between occupied nest sites of *Falco tinnunculus *** **in the study area in Vienna, Austria.**

The nearest neighbour distance (NND) decreases significantly with an increasing percentage of sealed soil (measured in a circle of radius r = 500 m around the nest site, Pearson Correlation, *N*_(2010)_ = 251, *r* = 0.47, *P* < 0.001, Figures 1 and 2). An analysis of microhabitat variables showed that the structure of buildings with nest sites differed significantly from those of buildings selected at random (Table 1). Unobstructed roof openings and the availability of green courtyards are more frequent at nest sites than at randomly chosen buildings. Accessible roof openings in buildings chosen at random are only found in the historical city centre with a soil sealing factor of more than 52%.

**Table 1:** **Habitat differences between buildings chosen at random (*N*** **= 240) and nest sites (*N*** **= 195) on buildings shown with a GLM with binomial error structure (random point = 0, nest site = 1) and a logit link function**

**Variable**	**Estimate**	***SE***	***T*-value**	***P*-value**	**Sign.**
Intercept	−3.11	0.70	−4.46	< 0.0001	***
Roof-openings [open = 1, closed = 0]	4.12	0.50	8.29	< 0.0001	***
Façade [smooth = 0, not smooth = 1]	−0.46	0.26	−1.79	0.07	•
Nest height/Height of the attic [m]	0.29	0.10	3.22	0.002	**
Green courtyard [yes = 1, no = 0]	0.88	0.27	3.33	<0.001	***

Significance codes: ‘***’ 0.001, ‘**’ 0.01, ‘•’ <0.1.

Unlike their conspecifics in some other European cities (e.g. [22,25,35]), kestrels temporarily leave Vienna during winter and return in spring. The dates when kestrels arrived at their nest sites differed only slightly along the urban gradient (Table 2, *P* = 0.06). In 2010, kestrels arrived at breeding sites in the city centre on average 3 days (±3.7 *SD*) earlier than at sites in suburban areas and in 2011 the difference was 7 days (±5.0 *SD*). Males usually occupied nest sites before females but the arrival dates of the two sexes overlapped.

**Table 2:** Dependence of breeding time (2010–2012) on the urban gradient (measured as percentage of sealed soil in r = 500 m around the nest site) and nearest neighbour distance (NND) as fixed effect in a generalized linear mixed model (GLMM)

**Breeding time**	**Estimate**	**SE**	***T*-value**	***Pr(>|t|)***	**expl.dev.(%)**	**Sign.**
**Arrival date**^**‡**^** ( *N * = 333)**						
Sealed soil	−12.36	6.47	−1.91	0.0568	54.74	•
NND^†^	−2.49	2.90	−0.86	0.3920	13.85	NS
(Intercept)	272.53	14.33	19.01	<0.0001		***
**Laying date**^**‡**^** ( *N * = 157)**						
Sealed soil	7.95	6.79	1.17	0.2440	40.68	NS
(Intercept)	28.53	4.86	5.85	<0.0001		***

The nest site ID and the study year were included as random factors. The error family was chosen according to the type of response variable as Gaussian family and identity link function. Explanatory deviance (in %) is given for each fixed effect.

*Note:*’^‡^’ data presented as residuals with the study year, ‘^†^’ log transformed.

Significance codes: ‘***’ 0.001, ‘•’ <0.1, ‘NS’ not significant.

### Breeding success and nestling survival along the urban gradient

There was no obvious effect of the urban gradient on the laying date (Table 2). The ratio of eggs hatched and the sizes of fledged broods depended upon the percentage of sealed soil and the laying date, both of which significantly decreased towards the city centre and for later broods (Table 3). Differences in urbanization and laying date were sufficient to account for 32% of the variance (*R*^*2*^ for GLMM) in breeding success (number of fledglings). The clutch size and the fledging rate were significantly influenced by the laying date, with fewer eggs and fewer fledged hatchlings in later nests (Table 3). The mean values and *SD* for the breeding data are given in Additional file 1.

**Table 3:** **Dependence of breeding parameters (2010–2012, *N*** **= 157) on the urban gradient (measured as percentage of sealed soil in r = 500 m around the nest site) as fixed effect in a generalized linear mixed model (GLMM)**

**Breeding parameter**	**Estimate**	**SE**	***Z*-value**	***Pr(>|z|)***	***R***^***2 ***^**for GLMM**	**Sign.**
**Clutch size ( *N * = 138)**					4.5	
Laying date^‡^	−0.01	0.00	−2.48	0.0132		*
(Intercept)	1.54	0.04	38.80	<0.0001		***
**Hatching rate**					15.44	
Laying date^‡^	−0.04	0.01	−2.94	0.0033		**
Sealed soil	−2.40	1.07	−2.23	0.0255		*
(Intercept)	2.54	0.78	3.24	0.0012		**
**Fledging rate**					16.04	
Laying date^‡^	−0.04	0.02	−2.06	0.0399		*
Sealed soil	−2.13	1.25	−1.71	0.0882		•
(Intercept)	2.60	0.99	2.62	0.0087		**
**Fledged brood size**					32.31	
Laying date^‡^	−0.02	0.00	−4.54	<0.0001		***
Sealed soil	−0.85	0.34	−2.48	0.0131		*
(Intercept)	1.26	0.25	5.04	<0.0001		***

The nest site ID and the study year were included as random factors. The error family was chosen according to the type of response variable.

*Note:*’^‡^’ data presented as residuals with the study year.

Significance codes: ‘***’ 0.001, ‘**’ 0.01, ‘*’ 0.05, ‘•’ <0.1.

**Table 4:** **Summary of model-selection according to Mark**[106]**for fixed-effects models of daily survival rate for kestrel nests (*N*** **= 157)**

**Model**	***K***	**AIC**_**c**_	Δ**AIC**_**c**_	***ω***_***i***_
Laying date^‡^ + Sealed soil (%)	3	271.42	0.00	0.5659
Laying date^‡^ + Sealed soil (%) + Age found	4	272.19	0.77	0.3852
Laying date^‡^	2	276.61	5.19	0.0422
Distance (m)^†^ from closest open green space (≥1 ha) + Sealed soil (%)	3	282.05	10.62	0.0028
Presence/absence of green courtyard + Sealed soil (%)	3	282.88	11.45	0.0018
Sealed soil (%)	2	283.86	12.44	0.0011
Age found + Sealed soil (%)	3	284.89	13.47	0.0007
Nearest neighbour distance (m)^†^	2	288.30	16.88	0.0001
Age found	2	290.46	19.04	0
Intercept-only model (constant daily survival rate)	1	290.49	19.07	0
Time Trend	2	290.89	19.47	0
Traffic area (m^2^, in r = 100 m around the nest site)^†^	2	291.27	19.85	0

*K* is the number of parameters in the model and *ω*_*i*_ the model weight.

*Note:*^‘‡’^ data presented as residuals with the study year, ^‘†’^ log transformed.

**Table 5:** **Number of nest attempts, reproductive outcome and cause of complete nest failure for *Falco tinnunculus *** **in Vienna, Austria 2010–2012**

		**Reproductive outcome**		**Time of nest failure**		**Cause of nest failure**		
Year	Nest attempts	Success (%)	Failure (%)	Egg stage	Nestling stage	Abandoned	Predation^#^	Other
2010	36	21 (58%)	15 (42%)	11	4	5	4	6
2011	52	36 (69%)	16 (31%)	14	2	4	6	6
2012	69	48 (70%)	21 (30%)	18	3	6	4	11
Total	157	105 (67%)	52 (33%)	43	9	15	14	23

*Note: ‘*^#^’ based on confirmed predation. If the predation event was not directly observed and the predator not identified, nest failure is assigned to other.

We found a primary sex ratio of 47% female and 53% male offspring (variation from hypothesized 1:1 ratio, *N* = 71 broods, exact binomial test 2011: *P* = 0.82; 2012: *P* = 0.22), whereas the sex ratio at fledging was 54% female and 46% male (*N* = 91 broods, 0.23 < *P* < 0.33). Female offspring have a slightly higher rate of survival; of the chicks lost as nestlings (*N* = 54 individuals), 31% were females and 69% were males (*χ*^*2*^ = 3.84, *P* = 0.05).

### Causes of nest failure

The initial fixed–effects model of nest survival included laying date and the percentage of sealed soil (Table 4). The best model shows daily survival rates decreasing with percentage of sealed soil from the suburbs towards the city centre and with later laying dates. As there was only a slight difference from the model that includes the age of the nestlings when the nest was found, we are confident that the results are not biased by when breeding was confirmed (during incubation or during the nestling phase). We tested for the influence of NNDs on nest failure, as reproductive performance is expected to decline with increasing population density, but the resulting model did not meet the criteria for good candidate models. To test tolerance against a potential anthropogenic stressor, we incorporated areas used by traffic in one model but in contrast to the observations on American kestrels (*Falco sparverius*[36]) we found no correlation.

A total of 33% of nests failed, with no statistically significant differences between years (Kruskal-Wallis *χ*^*2*^_*(2,157)*_ = 2.06, *P =* 0.36). 83% of nest failures occurred during incubation, with 27% of failures connected to predation as confirmed by direct observation (Table 5) and 29% due to nest desertion. Hooded crows (*Corvus cornix*) and Carrion crows (*Corvus corone*) are both common in Vienna but we found no significant difference in the abundance of these potential nest predators along the urban gradient (*Z* = 0.76, *P* = 0.45).

### Availability of prey

No significant relationship was found between abundance of prey and breeding success. Neither the number of prey-sized birds nor the abundance of rodents was able to predict the occurrence of successful breeders (GLM with proportion of successful nests per transect as dependent variable with binomial error distribution and logit link function [37] and both average numbers of birds and rodents as two predictors in the model, *N* = 25 transects, *P* for all predictors was not significant; birds: *Z* = 1.13, *P* = 0.25; rodents: *Z* = 0.42, *P* = 0.42).

The abundance of prey-sized species of bird varies with location along the urban gradient. No difference was found for thrush-sized birds (GLM with urban zone as predictor variable *Z* = 0.91, *P* = 0.36) but sparrow-sized birds were more abundant in suburban areas (*Z* = 11.08, *P* < 0.001) and pigeons – which our pellet analysis confirmed were included in kestrels’ diet – were more abundant in the city centre (*Z* = 3.49, *P* < 0.001).

The rodent survey included 2,676 trapping events (*N* = 129 individuals) and caught almost exclusively field mice of the genus *Apodemus* (98.4% of three species, *A. sylvaticus*, *A. flavicollis* and *A. uralensis*), with very small numbers of house mice *Mus musculus*, brown rats *Rattus norvegicus* and bank voles *Clethrionomys glareolus* recorded. In view of the relatively minor importance of field mice in the diet of urban kestrels (see below) and of the small sample size, an analysis of the trapping data by urban zone was not undertaken. Of the species trapped in the survey, only the bank vole is active by day [38], so the results indicate that diurnal rodents are hardly available in the city. The situation is in stark contrast to the surrounding areas, where diurnal voles (especially *Microtus arvalis*) are common [39,40].

### Diet choice in three urban zones

Pellet analysis showed no difference in the proportions of the main categories of prey between years (Kruskal-Wallis *χ*^*2*^-test: 0.22, *P* < 0.62). There were differences between urban zones: pellets in the city centre (*N* = 18 nest sites) consisted of 48.5% (by biomass, for details of calculation see Methods) mammals, 39% birds, 3.5% reptiles and 9% insects, while pellets found in the mixed zone (*N* = 10 nest sites) consisted of 56.6% mammals, 29.8% birds, 1.5% insects and 12.1% reptiles. The pellets found in suburban areas (*N* = 9 nest sites) showed 79.6% mammals, 12.2% birds, 4% insects and 4.2% reptiles. We could not identify all pellet contents to the species level but 70.4% of small mammals that could be identified were *Microtus arvalis* voles (sub-sample size: *N* = 152 individuals). Other mammal species identified were 13.0% field mice (*Apodemus* spp.) and 8.3% shrews (Soricidae).

The ratio of pairs that preyed mainly on mammals as opposed to on birds (based on the estimated biomass per nest site) differed significantly between urban zones (mammals: Kruskal-Wallis *χ*^*2*^_*(2)*_ = 7.54, *P* = 0.02 and birds: *χ*^*2*^_*(2)*_ = 7.24, *P* = 0.03), as did Levin’s index for breadth of diet, which was highest in the city centre (Kruskal-Wallis *χ*^*2*^_*(2)*_ = 8.34, *P* = 0.02; Levin’s index in the city centre: 4.02, mixed zone: 3.10 and suburban area: 1.44). Reptiles were preyed upon more often in the mixed zone (Kruskal-Wallis *χ*^*2*^_*(2)*_ = 5.67, *P* = 0.06), while insects were taken at approximately equal amounts in all urban zones (Kruskal-Wallis *χ*^*2*^_*(2)*_ = 0.61, *P* = 0.74).

## Discussion

### Choice and availability of nest site

Nearest neighbour distances (NND) decreased with increasing percentage of sealed soil (Figure 2) but pairs in the city centre had lower reproductive success, measured in terms of hatching rates and sizes of fledged broods, than pairs in suburban areas. As falcons do not construct nests themselves, their breeding locations are limited by the availability of potential nest sites [41,42]. The correlation between the number of nest sites and the number of roof openings (Table 1) supports the notion that more kestrels breed in the city centre due to the greater availability of building cavities. This can be attributed to the structural element of roof openings, which are limited to historical buildings in the city center.

Many species rely on environmental cues for a rapid assessment of habitat quality, thereby reducing the time and cost of finding a suitable breeding site [43-45]. In environments that have been altered, the use of cues that were formerly reliable might lead to reduced reproduction, turning these environments into ecological traps [19]. Most ecological traps have an anthropogenic origin [46] and migratory species might be more likely to fall into ecological traps created by urban landscapes [47]; compared to residents, migratory birds have more stringent time constraints in assessing the quality of breeding sites [44,48,49]. Early arriving individuals usually have preferential access to the best sites and partners, while later arrivals must settle in territories of progressively lower quality ([50,51]). For territorial birds such as the kestrel this should result in a sort of ideal-despotic distribution [52] where males first occupy the best sites, with poorer sites occupied successively later. We would expect the territories occupied first to show the highest breeding success but our study revealed the opposite to be the case. Kestrels breeding in the centre of Vienna tended to arrive before their suburban conspecifics (Table 2), suggesting that inner-city sites are assessed as being of at least equal quality. However, there were no differences in laying dates along the urban gradient and breeding performance (Table 3) was worse in inner-city districts than in the outskirts. Thus, the first returning kestrels do not select the best breeding sites. Breeding in highly urbanized areas was associated with higher rates of nest failure. Our models of nest survival showed that the percentage of sealed soil and the laying date are the main variables connected to nest failure (Table 4). A close proximity to large open green spaces (≥1 ha) and the presence of green courtyards also increased nest survival.

If highly urbanized areas are not associated with a breeding advantage, why are they occupied ahead of more productive sites at the edge of the city? It is possible that there are simply too few breeding cavities in the outskirts of the city. We found nest site cavities exclusively in the centre and conclude that closed breeding cavities are chosen because of their attractiveness and not because of the limited numbers of other potential types of nest, such as crow nests and window boxes. Attributes of breeding cavities such as limited accessibility to predators, protection from rain and sun and a low probability of collapse have been associated with higher breeding success [53,54]. Our study appears to show the opposite, with the selection of breeding cavities in the city centre associated with a lower breeding success.

### Nest failure, breeding success and sex-biased nestling survival

Most nest failures occurred during incubation of the eggs and were connected to nest desertion or predation (Table 5). Our results do not indicate a lower rate of nest predation for urban-breeding birds, as has been documented in other studies ([55,56] but see [57] reporting higher nest predation by corvids in urban areas). Abandonment occurred during the egg stage (once after hatching) and might have related to territorial disputes or to higher ectoparasite burdens in breeding cavities.

In common with many other raptors, the kestrel shows a size dimorphism, with females larger than males [58].When individuals of one sex are more costly than the other to produce, sex ratios may differ from 1:1 [59]. A higher mortality of the more expensive sex results in an excess of the cheaper sex at fledging and several species of raptor are known to manipulate the sex ratio of their offspring in response to a range of factors (e.g. [60-62]), including variation in the availability of resources [63,64]. Kestrels have been reported to switch the sex-bias from male-dominated in early nests to female-dominated in later nests [65]. We found that the smaller males and the last chicks to hatch were most likely to die as nestlings. The results are consistent with the finding that kestrels breeding in the centre of Warsaw had more female offspring [66]. The mortality of nestling Montagu’s harriers (*Circus pygargus*) has also been shown to be biased, with smaller males most likely to die, especially if they hatch later in the season [67]. Our results do not necessarily imply a manipulation of the sex ratio but could relate simply to a greater susceptibility of the smaller (male) chicks when food resources are scarce.

### Prey availability and diet choice

Rodents provide a higher nutritional value than avian prey [68,69]. Our survey of small mammals suggests that rodents are abundant in the city centre and the outskirts of Vienna but most species are nocturnal and thus hardly accessible to a diurnal raptor. Unlike the lesser kestrel *F. naumanni*, which is known to hunt during the night under artificial lighting [70], the kestrel is a largely diurnal hunter. Urban kestrels thus have to fly longer distances of at least several kilometres to hunt for their preferred prey [71,72]. In the centre of larger cities it may be energetically preferable to switch to less profitable but more common avian prey [73]. Indeed, recent studies indicate that kestrel populations in some larger European cities are increasingly feeding on birds [23,34,74], whereas kestrels in smaller or medium-sized European cities rely largely on a diet of voles (*Microtus* sp.), as do their rural conspecifics [24,25,72]. In general, kestrels are believed to feed on what is locally abundant, although there have been reports of consistent differences in diet composition between neighbouring breeding pairs, presumably reflecting individual preferences for prey or differing abilities at catching different prey types [75].

The increased proportion of non-rodent prey in kestrel pellets from the centre of Vienna compared with those from nearer the edge of the city is evidence that the birds generally hunt in the surroundings of their nest sites. Consistent with this idea, nest sites are often located close to green courtyards. A comparative study on generalist and specialist avian predators under fluctuating food conditions has shown that a vole specialist (pallid harrier *Circus macrourus*) forages less efficiently in poor vole years because the species is less efficient at capturing alternative prey, such as birds [76]. The increased effort required to hunt non-rodent prey may affect the breeding success of kestrels in the centre of Vienna. Our data indicate a trade-off between the ready availability of breeding cavities and the greater distances to hunting grounds, which result in a shift in the main prey taken and a lower breeding success.

### Are inner-city buildings ecological traps for an urban raptor?

The kestrel is not truly an urban species. Although it has a strong preference for breeding in cavities, it does not profit from other human resources, nor does it show a higher degree of sociality and sedentariness [77]. It clearly exploits the urban environment but high breeding densities in human-dominated landscapes do not necessarily indicate that the species benefits in terms of breeding success. Our findings are consistent with a trade-off between the availability of building cavities, which offer nest sites that are protected from potential predators, and the poorer food supply in the city centre. The consequence is that kestrels appear to select nest sites that are associated with increased reproductive failure and smaller fledged broods.

It may be difficult for kestrels to evaluate food availability when they are prospecting for nest sites ([78,79] and citations therein) and errors could cause birds to overestimate the quality of the habitat [78,80] and settle in poor habitats despite the availability of better options. The preference for poorer habitats is a maladaptive behaviour associated with so-called ecological traps (reviewed in [19,43,46,47,81]). The idea that kestrels are falling into an ecological trap should be further investigated as it could be of conservation concern and might have important consequences for the viability of certain populations.

## Conclusions

In the centre of Vienna, Austria, kestrels frequently breed in roof openings in historical buildings, a structural feature that is not available in the outskirts of the city. A comparison along the urban gradient shows the smallest nearest neighbour distances for pairs that breed in the city centre. The kestrel’s favoured prey is rodents but in the centre rodents are less abundant and largely nocturnal and thus not available to diurnally hunting raptors. Kestrels breeding in the centre of Vienna consume more birds, including pigeons, and fewer rodents than kestrels in the outskirts. The city-dwelling raptor pays a high price for life in the city, with a lower reproductive success than birds breeding in the outskirts. The kestrel might appear to be an urban exploiter but given the poor reproductive performance of urban kestrels it is likely that the species is falling into an ecological trap. Although the kestrel is not itself of conservation concern, our findings suggest that other city-dwelling species may be faring less well than their abundance in the urban environment would appear to indicate.

## Methods

### Study system

The Eurasian kestrel, hereafter simply referred to as the kestrel, is the most abundant raptor in Vienna, Austria (48°12’N, 16°22’E; 415 km^2^, ca. 150 – 500 m a. s. l., 1.8 million inhabitants). The estimated population density of 60–96 breeding pairs per 100 km^2^[32] is high compared to that in other European metropolises (e.g. [82,83]) and in rural eastern Austria [84]. Kestrels return to Vienna at the end of March, before pair formation, and remain at their breeding sites until late summer (pers. obs. PS and AG). The study period covered three breeding seasons from March 2010 to August 2012.

The river Danube, lined with riparian forest, divides Vienna in two, making distance from the city centre misleading in terms of defining an urban gradient. We thus define urbanization by the percentage of sealed soil (calculated in ArcGIS 10 by ESRI ©, based on land covered by buildings or areas used by traffic on a land allocation map, digitized in 55 categories of land utilization between 2007 and 2010, in a circle of radius 500 m around the nest sites; *sensu*[85]). Areas with < 1% of unsealed soil were defined as rural and excluded from the analysis. Excluding these surroundings, mostly forested and agricultural areas, the urban study area covered 243 km^2^ (Figure 1). Nests were distributed between percentages of sealed soil of 18% (most suburban) and 89% (most urban). By extending our search up to 1% soil sealing we made sure that NNDs were accurate.

With the help of local media we called on the public to report kestrel nests in Vienna in 2010 and 2011. Additionally, 25 volunteer ornithologists and PS and AG systematically searched the city for nests. Historic nest sites recorded in the BirdLife Austria archive (*N* = 103), occupied nests found during systematic searches (*N* = 124), locations of kestrel foundlings in the database of the animal shelter and the bird clinic at the University of Veterinary Medicine, Vienna (*N* = 78) and nest sites reported by the general public were confirmed through personal observations during pair formation and courtship and classified as occupied if adults were present on two consecutive visits. During the study period we built a data base of 451 recent nest sites, between 50% and 65% of which were occupied each year.

### Nest site and habitat parameters

Two different spatial levels were used to define nest site and habitat parameters. The percentage of sealed soil was calculated in a circle of r = 500 m around the nest site (78.5 ha) and expressed as the percentage of land covered by buildings or areas used by traffic. The resulting value is termed the urban gradient. The distance (in m) from the nest site to the nearest open green space was recorded. The size of the nearest open green space, which was either a green courtyard or a park area in the city centre or a lawn (usually in a garden), a meadow or agricultural land in the suburbs, was assigned to one of four categories, ≥ 1 ha, ≥ 0.5 ha, ≥ 0.25 ha and ≥ 100 m^2^.

We also described the building on which the nest was located, recording the nest height (m), façade structure, presence of roof openings or other cavities and presence of green courtyards (between 0.01 and 0.1 ha). We counted the stick nests of crows on the façade and in surrounding trees, as well as the number of window boxes on balconies. The same parameters were measured for 240 buildings chosen at random by placing a 500×500 m grid over the study area and using each intersection that touched a building. We used the height of the attic as hypothetical ‘nest height’ variable (as 62% of actual nest sites were located at attic level).

Habitat data were obtained via a land allocation map (1:7,500, resolution 15 cm), digitized based on geo-referenced aerial images provided by the Environmental Protection Bureau of Vienna (MA22-709/2010). Data on building structure were acquired on site.

### Breeding parameters

Occupied nests that were accessible via the attic or by climbing were monitored 4–6 times during each breeding season to determine (1) the laying date, (2) the clutch size, (3) the number of hatched offspring and (4) the number of fledged young. In total, 157 broods were examined (36 nest sites in 2010, 52 in 2011 and 69 in 2012). Kestrels start incubation after the second egg is laid and the date (variable ‘laying date’) was estimated either directly or by subtracting 30 days from the estimated date of hatching [58]. We defined 1 April as day 1 of the breeding season and numbered all dates of nest inspection thereafter for analysing survival (in total 118 days, see [86] for methodological details). We used the residuals of laying date and study year (calculated in an ANOVA with study year as predictor and laying date as predicted variable) to compare differences along the urban gradient. Additional covariates for nest survival models were percentage of sealed soil (%), age at which the nest was found, distance (m) from the closest open green space (area ≥ 1 ha) as a potential large hunting ground, presence/absence of a green courtyard (between 0.01 and 0.1 ha) with in r = 100 m from the nest site (factor variable 1/0) as a potential small hunting ground, area used by traffic (m^2^, in a circle of r = 100 m around the nest site) as an indicator of noise disturbance and the NND (m) to the next active kestrel nest. In two years we additionally recorded for a larger data set (*N* = 200 nests in 2010 and *N* = 185 nests in 2011) the dates kestrels arrive at their nest sites: the information was provided by ornithologists involved in the breeding bird survey and observers living in direct view of a nest site. Involving the general public allowed us to have observers at accessible nest sites (mostly across the street or ‘owners’ of occupied window and nest boxes), who provided immediate information on hatching. In other cases we estimated the date of hatching from clutch initiation or egg floating. We marked chicks after hatching with non-toxic ink until they were ringed.

During repeated monitoring, the nestlings were measured, weighed and ringed (with rings from the Ringing Centre Radolfzell, Germany) when they were at least 10 days old (wing length ≥ 54 mm). The lengths of the culmen, tail, wing, tarsus, claws and feet [87] were measured for age determination [21]. We determined clutch size, hatching and fledging rates and size of the fledged brood (breeding success) for each nest. The hatching rate was recorded on a continuous scale from 0 (no eggs hatched) to 1 (all eggs hatched). The fledging rate was defined similarly and varied from 0 (no hatchling survived) to 1 (all hatched young successfully fledged). The final inspection took place in the last week of the nestling period (24–30 days after hatching). Nestlings fledge after 28–31 days [58], so we considered pairs successful if they produced at least one 28-day-old chick. The size of the fledged brood was therefore the number of nestlings in successful nests at week 4.

Nests were defined as having failed if there was clutch loss during incubation or if all chicks died after hatching (as a result of predation, starvation, parasite infestation or parental abandonment). We attributed the cause of failure to abandonment if the nest contained intact and cold eggs and no adults were present during two subsequent inspections over 1–2 weeks (*sensu*[36]) and to predation if predation was observed (crows robbing the nest during the day or broken eggs and marten tracks found in the breeding niche).

### Sexing chicks

Sexing of chicks was based on the CHD system, Intron A [88]. We used the blastoderm or embryonic tissue from unhatched eggs, buccal swabs [89] for small nestlings (2–10 days) and blood of pinned growing feathers for older nestlings (>10 days). DNA was extracted with the QIAGEN DNeasy Blood & Tissue Kit following the standard protocol with Proteinase K. Sex was determined based on the 2718R and 2550 F primer set [90] and confirmed with the *Falco*-specific *fp*102 and *fp*49 primers [91]. PCR amplification was performed in 25 μl containing 0.5 μl 10 mM dNTP, 0.25 μl of each forward and reverse primer (50 pmol/μl), 0.25 μl Dynazyme Polymerase and 2.5 μl 10x reaction buffer. PCR was performed with 40 cycles of 2 min at 94°C, 20 s at 50°C and 40 s at 72°C followed by 5 min at 72°C. PCR products were visualized on 2% agarose gels. The primary sex ratio was defined as the sex ratio in the full clutch (recorded in 2011 and 2012). The secondary sex ratio was defined as the sex ratio at fledging (recorded in all years).

### Pellet analysis and abundance of prey

In 2010 and 2011, 637 pellets and remains of prey were collected from 37 different nest sites. We grouped the findings at nest sites according to their location along the urban gradient (*sensu*[34]), distinguishing between city centre (288 pellets, *N* = 18 nests with 81-89% sealed soil), mixed zone (206 pellets, *N* = 10 nests, 51-80% sealed soil) and suburban areas (143 pellets, *N* = 9 nests, 18-50% sealed soil). The pellets were dissected and prey remains classified as ‘mammals’, ‘birds’, ‘reptiles’ or ‘insects’. We identified prey to species level where possible with the aid of reference collections at the Museum of Natural History, Vienna. We assessed the minimum number of each category of prey per pellet (largest number of different jaws, upper or lower mandibles, skulls or pairs of incisors in small mammals; plugged feathers in birds; pairs of mandibles, tarsi or ovipositors in insects) and present data as their estimated biomass [g]: 18.8 g for small mammals, 22.4 g for sparrow-sized birds, 76.4 g for thrush-sized birds, 330 g for pigeons, 10 g for reptiles, 1.5 g for Orthoptera and 0.2 g for Coleoptera [92,93]. Diet breadth (B) was calculated according to Levin’s index [94] as B = 1/Σp_i_^2^, where p_i_ is the proportion of the diet represented by prey type i. As variables were not normally distributed, nonparametric tests were used for analysis.

To assess the availability of potential avian prey in 2010, a team of 25 ornithologists monitored 25 transects (*N* = 9 in the city centre, *N* = 9 in the mixed zone and *N* = 7 in suburban areas) in the course of the Austrian breeding bird survey using the standard method of 5-minute point-counts in the early morning under stable weather conditions [95]. The ornithologists were recruited by Birdlife Austria and by PS. Each bird recorded within 50 m of the point was identified based on voice and/or appearance. Analysis was based on prey known from pellet analysis [33] to be taken by kestrels. Potential prey was grouped by size (sparrow-, thrush- and pigeon-sized). Transects were selected by PS in ArcGIS 10 based on the land allocation map and included buildings, areas used by traffic, green courtyards (between 0.01 and 0.1 ha) and parks (between 0.3 and 600 ha) in the city centre and the mixed zone, and gardens and forest edges in the suburban area. Transects were chosen independently of the location of kestrel nests. They were sampled twice per year, at the beginning of the breeding season (in spring, calendar week 17–18, in April) and during the nestling period (in summer, calendar week 22–23, in June). Each transect consisted of 12–20 points at 300–500 m intervals.

The kestrel nest sites were assigned to the closest transects (max. distance 1 km, *N* = 2-24 nests/transect). It is logical to allocate a nest to a transect rather than to a point as two or more count points could be within the hunting grounds of a single pair of kestrels. Furthermore, the assignment takes into account the spatial autocorrelation of neighbouring counting points on a transect. The proportion of successful breeding attempts was calculated for each transect and the figures were used to relate breeding success to availability of prey.

Densities of rodents were estimated by means of the ‘minimum number alive method’ of [96]. We used 97 Rödl-type live traps [97] in 59 transects, with 10–20 traps in each of 23 different city parks (between 0.3 and 600 ha) across the urban gradient. The traps were checked twice per day (morning and evening) on two consecutive days per area at the start and the end of the 2010 breeding season, resulting in 2,676 trapping events (see [98] for details).

### Statistical analysis

Differences in habitat between nest sites and buildings chosen at random were evaluated with a generalized linear model (GLM) with binomial error structure and a logit link function. The variables were nest height, facade structure, presence of roof openings or other cavities, and presence of green courtyards. One variable, houses with alcoves, was excluded because there were more roof openings in houses with alcoves (χ^*2*^-test, *N* = 248, *df* = 1, *P* < 0.001) and the variable ‘roof openings’ was obviously related to nest site and thus of higher biological significance.

To analyse the relationship between abundance of prey and breeding success, a GLM was constructed with proportion of successful nests as dependent variable and the two predictors ‘avian prey counted’ and ‘rodents trapped’. To calculate the proportion of successful nests we used the number of successful and failed nests per transect together as response variable fitted to a binomial error distribution. This can be treated as a weighted regression using the individual sample sizes as weights and the logit link function to ensure linearity (see [37] for details).

All distance and area variables were logarithmically transformed. Analysis of the variation of breeding parameters with the urban gradient was performed by generalized linear mixed models (GLMM) with the lmer and glmer functions of the R package ‘lme4’ [99], including the nest site ID and the study year as random factors. Error distribution was chosen according to the response variable: Gaussian distribution and the identity link function for clutch date and date of arrival at the nest site; binomial distribution and the logit link function for rates of hatching and fledging (values between 0 and 1); and Poisson distribution with the log link function for the sizes of the clutch and the fledged brood.

Models including soil sealing (urban gradient), NND (nearest neighbour distance) and laying date (timing of breeding) as explanatory variables were evaluated, as was a model including interactions between these variables. All explanatory variables were fitted to a maximal model and removed one by one, with the associated changes in the model deviance assessed by a likelihood ratio test [100]. After each step we calculated the AIC_c_ (Akaike Information Criterion, corrected for small sample sizes) and defined the model with the lowest value as the final one [101]. Model selection and model weight is presented in Additional file 2. The proportion of deviance explained (%) for each fixed effect of the lmer models was analysed with the ‘LMER Convenience Functions’ package [102]. As this function has not yet been implemented for glmer models (lme4 requires binomial and Poisson error distributions) we assessed estimates of variance explained using *R*^*2*^ values, following the method recently described by [103], implemented in the ‘MuMIn’ package [104]. Details on nest site and habitat parameters used for statistical analysis can be found in Additional file 3. To analyse nest survival we used the ‘nest’ model in ‘RMark’ [105,106]. We considered models with ΔAIC < 2.0 to represent good candidates [107]. All statistical analysis was performed with the software R version 3.0.1 (R Development Core Team 2013).

### Ethical notes

The study was performed under license from the Ethics Committee of the University of Veterinary Medicine, Vienna and the Environmental Protection Bureau of Vienna (MA 22/1263/2010/3). All sampling was conducted in strict accordance with current Austrian and EU law and followed the Weatherall Report and the guidelines for the treatment of animals in behavioural research and teaching [108].

### Availability of supporting data

Morphological data on kestrels have been provided to the Ringing Centre in Radolfzell, Germany. Data from the breeding bird survey have been made available to Birdlife Austria and the Environmental Protection Bureau of Vienna (MA22) for use in conservation measures. All supporting data are available from the authors on request.

## Abbreviations

AICc: Akaike information criterion, corrected for small sample sizes; GLM: Generalized linear model; GLMM: Generalized linear mixed; NND: Nearest neighbour distance.

## Competing interests

The authors declare that they have no competing interests.

## Authors’ contributions

The original idea and study design came from PS, AG and HWK. PS performed the field and laboratory work; help by others is accordingly acknowledged. PS, EN and GT analysed the data. The manuscript was prepared by PS, EN, GT and AG and approved by all authors.

## Supplementary Material

Additional file 1 **Breeding parameters of *Falco tinnunculus *** **in Vienna, Austria, 2010-2012 (*N *** **= 157 nest sites in total) in three urban zones.** Results are shown as mean value ± *SD.* We pooled those nest sites according to their location along the urban gradient (city centre with 81%-89% soil sealing, mixed zone with 51-80% soil sealing, and suburban area with 18-50% soil sealing). Click here for file

Additional file 2 **Model selection for Table **3** in results section (dependence of breeding parameters on urbanization).** Models are ranked according to the Akaike Information Criterion, corrected for small sample sizes (AIC_c_). The ΔAIC_c_ indicates AIC_c_ differences between a particular model and the best-fitting model with the smallest AIC_c_. Akaike weights (*ω*_*i*_) indicate the contribution of each model to the average of all candidate models and *K* the number of parameters. Variables included in and excluded from a particular model are indicated by 1s and 0s, respectively. ld – laying date, ss – sealed soil, NND – nearest neighbour distance. Good candidate models are printed in bold. Click here for file

Additional file 3 Nest site and habitat parameters used for statistical analysis. Click here for file
