# Supplementary material for: Hard times in the city – attractive nest sites but insufficient food supply lead to low reproduction rates in a bird of prey
Source: Front Zool. 2014 May 27;11:48. doi: 10.1186/1742-9994-11-48 (PMC4035672; doi:10.1186/1742-9994-11-48)
Supplement: Additional file 1 — Breeding parameters of Falco tinnunculus in Vienna, Austria, 2010-2012 ( N = 157 nest sites in total) in three urban zones. Results are shown as mean value ± SD. We pooled those nest sites according to their location along the urban gradient (city centre with 81%-89% soil sealing, mixed zone with 51-80% soil sealing, and suburban area with 18-50% soil sealing). [file 1742-9994-11-48-S1.docx]

**Additional File 1**: Breeding parameters of *Falco tinnunculus* in Vienna, Austria, 2010-2012 (*N*=157 nest sites in total) in three urban zones. Results are shown as mean value ± *SD.* We pooled those nest sites according to their location along the urban gradient (city centre with 81%-89% soil sealing, mixed zone with 51-80% soil sealing, and suburban area with 18-50% soil sealing).

|  | City centre | Mixed zone | Suburban area |
| --- | --- | --- | --- |
| 2010 (*N*=36, in total 251 occupied nests within the urban study area) | | | |
| Laying date (first egg) | May 4 ± 6.3 d (April 27) | May 3 ± 11.9 d (April 15) | Mai 1 ± 17.6 d (April 11) |
| Clutch size | 2.52 ± 2.06 | 4.58 ± 1.73 | 5.00 ± 1.41 |
| Hatched | 1.74 ± 1.94 | 3.58 ± 1.78 | 4.40 ± 1.14 |
| Fledged per breeding attempt | 1.00 ± 1.33 | 1.58 ± 1.31 | 4.00 ± 1.22 |
| Fledged per successful pair | 2.38 ± 0.92 | 2.38 ± 0.74 | 4.00 ± 1.22 |
| % successful pairs | 42.11% (*N*=8) | 66.67% (*N*=8) | 100.00% (*N*=5) |
| 2011 (*N*=52, in total 297 occupied nests) | | | |
| Laying date (first egg) | May 4 ± 14.4 d (April 7) | May 3 ± 15.1 d (April 6) | April 19 ± 7.2 d (April 8) |
| Clutchsize | 3.88 ± 1.86 | 4.46 ± 1.48 | 5.75 ± 1.16 |
| Hatched | 2.38 ± 2.42 | 3.57 ± 1.89 | 4.25 ± 2.71 |
| Fledged per breeding attempt | 1.81 ± 1.94 | 2.61 ± 1.79 | 3.50 ± 2.39 |
| Fledged per successful pair | 3.22 ± 1.39 | 3.32 ± 1.29 | 4.67 ± 1.21 |
| % successful pairs | 56.25% (*N*=9) | 78.57% (*N*=22) | 75.00% (*N*=6) |
| 2012 (*N*=69, in total 215 occupied nests) | | | |
| Laying date (first egg) | May 4 ± 11.3 d (April 12) | May 4 ± 15.8 d (April 5) | April 24 ± 16.42 d (April 4) |
| Clutch size | 3.47 ± 2.45 | 4.42 ± 1.65 | 5.00 ± 0.93 |
| Hatched | 2.83 ± 2.21 | 3.58 ± 2.11 | 4.13 ± 1.88 |
| Fledged per breeding attempt | 2.48 ± 1.95 | 2.81 ± 1.99 | 3.53 ± 2.10 |
| Fledged per successful pair | 3.80 ± 1.21 | 3.95 ± 0.95 | 4.42 ± 1.16 |
| % successful pairs | 65.22% (*N*=15) | 70.97% (*N*=22) | 80.00% (*N*=12) |
