# Supplementary material for: Hard times in the city – attractive nest sites but insufficient food supply lead to low reproduction rates in a bird of prey
Source: Front Zool. 2014 May 27;11:48. doi: 10.1186/1742-9994-11-48 (PMC4035672; doi:10.1186/1742-9994-11-48)
Supplement: Additional file 2 — Model selection for Table 3 in results section (dependence of breeding parameters on urbanization). Models are ranked according to the Akaike Information Criterion, corrected for small sample sizes (AICc). The ΔAICc indicates AICc differences between a particular model and the best-fitting model with the smallest AICc. Akaike weights (ω i ) indicate the contribution of each model to the average of all candidate models and K the number of parameters. Variables included in and excluded from a particular model are indicated by 1s and 0s, respectively. ld – laying date, ss – sealed soil, NND – nearest neighbour distance. Good candidate models are printed in bold. [file 1742-9994-11-48-S2.docx]

**Additional File 2**: Model selection for Table 3 in results section (dependence of breeding parameters on urbanization). Models are ranked according to the Akaike Information Criterion, corrected for small sample sizes (AIC_c_). The ΔAIC_c_ indicates AIC_c_ differences between a particular model and the best-fitting model with the smallest AIC_c_. Akaike weights (*ω_i_*) indicate the contribution of each model to the average of all candidate models and *K* the number of parameters. Variables included in and excluded from a particular model are indicated by 1s and 0s, respectively. ld – laying date, ss – sealed soil, NND – nearest neighbour distance. Good candidate models are printed in bold.

| **Table 3** | Variables included | | | Model selection based on AICc | | | |
| --- | --- | --- | --- | --- | --- | --- | --- |
| **Clutch size** | ld^‡^ | NND^†^ | ss | *K* | AIC_c_ | ΔAIC_c_ | *ω_i_* |
| Final model | **1** | **0** | **0** | **4** | **516.00** | **0** | **0.40** |
|  | **1** | **0** | **1** | **5** | **517.00** | **1.02** | **0.24** |
|  | 1 | 1 | 0 | 5 | 518.10 | 2.10 | 0.14 |
|  | 1 | 1 | 1 | 6 | 518.90 | 2.95 | 0.09 |
|  | 0 | 0 | 0 | 3 | 520.00 | 4.08 | 0.05 |
|  | 0 | 0 | 1 | 4 | 520.30 | 4.34 | 0.05 |
|  | 0 | 1 | 0 | 4 | 522.10 | 6.15 | 0.02 |
| Full model | 0 | 1 | 1 | 5 | 522.40 | 6.46 | 0.02 |
| **Hatching rate** | ld^‡^ | NND^†^ | ss | *K* | AIC_c_ | ΔAIC_c_ | *ω_i_* |
| Final model | **1** | **0** | **1** | **5** | **187.00** | **0** | **0.61** |
|  | 1 | 1 | 1 | 6 | 189.10 | 2.05 | 0.22 |
|  | 1 | 0 | 0 | 4 | 190.40 | 3.41 | 0.11 |
|  | 1 | 1 | 0 | 5 | 192.50 | 5.49 | 0.04 |
|  | 0 | 0 | 1 | 4 | 194.20 | 7.22 | 0.02 |
|  | 0 | 1 | 1 | 5 | 196.30 | 9.25 | 0.01 |
|  | 0 | 0 | 0 | 3 | 199.50 | 12.44 | 0 |
| Full model | 0 | 1 | 0 | 4 | 200.80 | 13.76 | 0 |
| **Fledging rate** | ld^‡^ | NND^†^ | ss | *K* | AIC_c_ | ΔAIC_c_ | *ω_i_* |
| Final model | **1** | **0** | **1** | **5** | **117.20** | **0** | **0.27** |
|  | **1** | **0** | **0** | **4** | **117.60** | **0.38** | **0.23** |
|  | **0** | **0** | **1** | **4** | **118.60** | **1.40** | **0.14** |
|  | **1** | **1** | **1** | **6** | **118.80** | **1.63** | **0.12** |
|  | 0 | 0 | 0 | 3 | 119.50 | 2.35 | 0.08 |
|  | 1 | 1 | 0 | 5 | 119.60 | 2.46 | 0.08 |
|  | 0 | 1 | 1 | 5 | 120.50 | 3.30 | 0.05 |
| Full model | 0 | 1 | 0 | 4 | 121.70 | 4.49 | 0.03 |
| **Fledged brood size** | ld^‡^ | NND^†^ | ss | *K* | AIC_c_ | ΔAIC_c_ | *ω_i_* |
| Final model | **1** | **0** | **1** | **5** | **628.80** | **0** | **0.47** |
|  | **1** | **1** | **1** | **6** | **629.00** | **0.25** | **0.41** |
|  | 1 | 0 | 0 | 4 | 632.20 | 3.46 | 0.08 |
|  | 1 | 1 | 0 | 5 | 633.70 | 4.97 | 0.04 |
|  | 0 | 0 | 1 | 4 | 647.90 | 19.14 | 0 |
|  | 0 | 1 | 1 | 5 | 649.40 | 20.69 | 0 |
|  | 0 | 0 | 0 | 3 | 654.00 | 25.21 | 0 |
| Full model | 0 | 1 | 0 | 4 | 656.10 | 27.31 | 0 |

*Note:* ‘^‡^’ data presented as residuals with the study year,‘^†^’ log transformed
