# Supplementary material for: Hard times in the city – attractive nest sites but insufficient food supply lead to low reproduction rates in a bird of prey
Source: Front Zool. 2014 May 27;11:48. doi: 10.1186/1742-9994-11-48 (PMC4035672; doi:10.1186/1742-9994-11-48)
Supplement: Additional file 3 — Nest site and habitat parameters used for statistical analysis. [file 1742-9994-11-48-S3.docx]

**Additional File 3**: Nest site and habitat parameters used for statistical analysis.

| **Habitat parameters** | **Detailed description** |
| --- | --- |
| Urban gradient | percentage of sealed soil (%), based on land covered by buildings or areas used by traffic calculated on a land allocation map (1:7,500, resolution 15 cm), digitized in 55 categories of land utilization between 2007 and 2010, in a circle of radius 500 m around the nest sites and random points  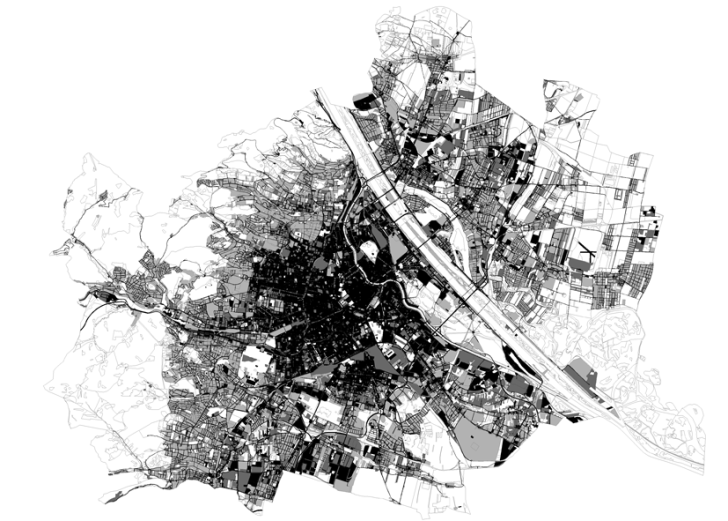 |
| City centre | 81%-89% sealed soil |
| Mixed zone | 51-80% sealed soil |
| Suburban area | 18-50% sealed soil |
| NND | m, nearest neighbour distance to the closest active kestrel nest |
| Distance to nearest open green space | m, assigned to four different size categories, ≥ 1 ha, ≥ 0.5 ha, ≥ 0.25 ha, ≥ 100 m^2^ |
| Traffic area | m^2^, measured in a circle of radius 100 m around the nest site as an indicator for noise disturbance  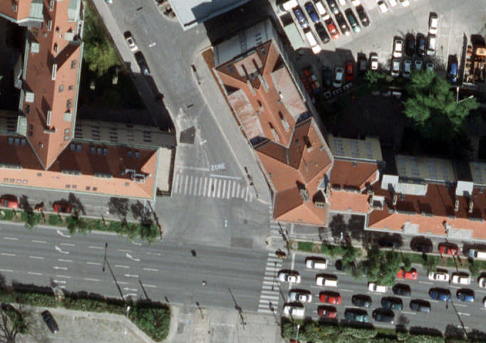 |

| **Nest site parameters** | **Detailed description** |
| --- | --- |
| Height | m, height of the nest site or height of the attic as hypothetical ‘nest height’ variable (as 62% of actual nest sites were located at attic level) |
| Facade structure | presence/absence of stucco work  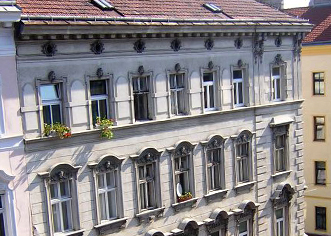 |
| Roof openings | presence/absence of specific architectural element (generally on buildings dating from pre-1940, especially from the so-called ‘Gründerzeit’ between 1848 and 1873) in the historic districts of Vienna, located between the highest row of windows and the roof; 24-62 cm in width, 16-50 cm in depth and 24-48 cm in height  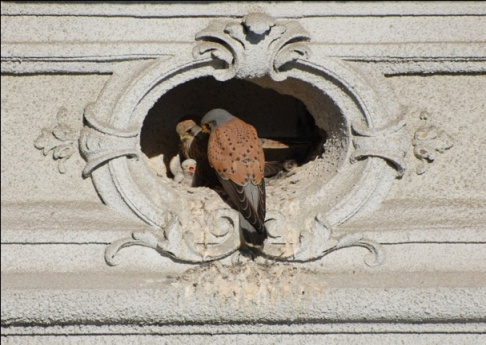 |
| Other building cavities | presence/absence, matching the size of a suitable breeding cavity  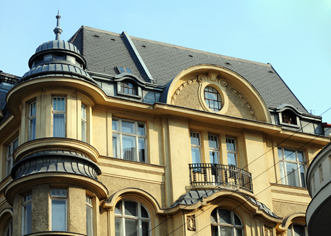 |
| Green courtyard | presence/absence, size between 0.01 and 0.1 ha  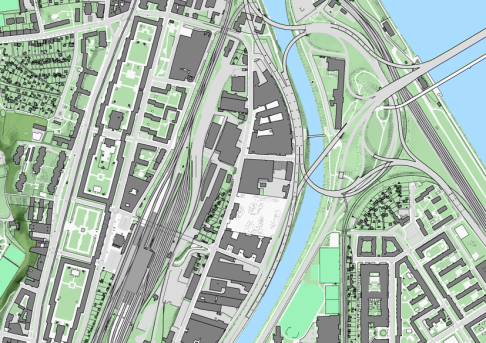 |
